# Thaw, biopsy and refreeze strategy for PGT-A on previously cryopreserved embryos

**Published:** 2020-01-24

**Authors:** M Wilding, M Terribile, I Parisi, G Nargund

**Affiliations:** Create fertility, 150 Cheapside, LondonEC2V 6ET, United Kingdom.

**Keywords:** PGT-A, aneuploidy, embryo biopsy, embryo refreezing, embryo cryopreservation, embryo quality

## Abstract

**Background:**

Preimplantation genetic testing for aneuploidy (PGT-A) with Next Generation Sequencing technology is a low-cost and powerful technology for the analysis of embryo quality. However, PGT-A requires freezing of embryos, suggesting that previously cryopreserved embryos cannot be tested. Here, we test whether use of the vitrification technique permits the refreezing of embryos, thus permitting PGT-A testing of cryopreserved embryos.

**Methods:**

The results are a retrospective analysis of cases performed at Create fertility between 2016 and 2017. Results obtained after traditional PGT-A are compared with results after the thaw biopsy and refreeze (TBR) procedure. A total of 220 patients were treated with PGT-A and 54 patients with the TBR procedure.

**Results:**

Maternal ages were not significantly different between the groups. The proportion of PGT-A normal embryos was not significantly different between the two groups. A clinical pregnancy rate of 61.5% was achieved with the PGT-A group and 52.4% with the TBR group. These results were not statistically significant. The efficiency of the thaw, biopsy and refreeze technique was not significantly different to that of fresh cycles for rates of survival, results obtained and aneuploidy incidence. Clinical pregnancy rates are not significantly different after the biopsy of fresh and previously cryopreserved embryos.

**Conclusion:**

The data shows that the TBR procedure has an equivalent success rate to that of classical PGT-A procedures.

## Introduction

The technique of preimplantation genetic testing for aneuploidies (PGT-A) enables the selection of chromosomally normal human embryos for replacement into the uterus (Cinnioglu, 2019). The aim of PGT-A is to improve the methods of section of embryos for transfer and therefore decrease the time to live birth (Kang et al., 2016; Cinnioglu, 2019). Results obtained with PGS have improved markedly through the application of new technology for the analysis of biopsied samples (Dahdouh et al., 2015a,b; Coates et al., 2017; Friedenthal et al., 2018; Fragouli, 2018; Cinnioglu, 2019). Results suggest that PGT-A is cost-effective in patients over 37 years old (Goldman et al., 2018; Collins et al., 2017).

Next generation sequencing (NGS) is a powerful new technology that has enabled low- cost sequencing of entire genomes with increased sensitivity (Fraguoli, 2018). NGS is now becoming the technique of choice for PGT-A (Fraguoli, 2018). However, the extended timeframe for NGS results (2-3 weeks) does preclude embryo transfer in the same cycle as embryo testing. Embryo cryopreservation techniques are therefore required.

Patients wishing to have PGT-A on previously cryopreserved embryos are currently limited from accessing this technique due to the requirement for refreezing of the embryos. Embryo cryopreservation techniques have, however, significantly improved in the recent years due the introduction of the vitrification method (Yokota et al., 2000).

Although refreezing of human embryos with the vitrification method has been reported in the literature (Yokota et al., 2001; Smith et al., 2005; Farhat et al., 2001; Kumsako et al., 2009; Ludwig et al., 1999; Murakami et al., 2011; Lierman et al., 2014), reports are rare and the refreezing of embryos is generally considered to be a technique to be used only in emergency situations (Kumasako et al., 2008). Despite this, human refrozen embryos have been transferred to patients with successful outcomes (Farhat et al., 2001; Safari et al., 2016; Batwala et al., 2018).

Previously, we and others have shown that the vitrification of human embryos after slow-thawing is practical for analysis with PGT-A (Grifo et al., 2013; Batwala et al., 2018). In this report, we compare the results obtained from embryos thawed, biopsied and refrozen (TBR) with embryos biopsied for PGT-A without a prior freeze. The results show that the use of the thaw, biopsy and refreeze technique gives comparable results to PGT-A on fresh embryos and demonstrate that the technique is suitable for general use in the laboratory of assisted reproduction.

## Materials and methods

### Study type

The study is a proof of concept retrospective analysis of results obtained from patients attending Create fertility between 2016 and 2017. Patients elected to have a cycle of assisted reproduction with PGT-A testing of freshly produced embryos, or with previously cryopreserved embryos. All patients were counselled and signed informed consent for the procedures reported in this work. Furthermore, the refreeze technique has been previously published (Grifo et al., 2013; Smith et al., 2005; Farhat et al., 2001; Kumsako et al., 2009; Ludwig et al., 1999; Lierman et al., 2014).

### Patients

Patients were attending Create fertility, St Pauls, London for assisted reproduction treatment. Couples were included if the female menstrual cycle ranged 24-35 days (intra-individual variability +/- 3 days), if the karyotype of both subjects of the couple was normal and if biochemical assessments demonstrated the absence of metabolic, autoimmune and infectious disorders. Patients were excluded from the program if the female body mass index (BMI=weight (kg)/height (m)2 x100) was > 29, if biochemical and/or USG evidence suggested polycystic ovarian syndrome, if the female partner had stage III-IV endometriosis, if autoimmune, thyroid or chromosomal abnormalities were present and if only one ovary was present. Couples in which the semen sample concentration was < 1 million/ ml or derived from either a cryopreserved sample or surgical retrieval techniques were also excluded to prevent a male-factor bias. Patients were prepared using standard controlled mild ovarian hyperstimulation protocols including ovarian stimulation with exogenous FSH (Bemfola, Gedeon Richter, Budapest, Hungary) and prevention of premature ovulation with a GnRH antagonist (Cetrotidex acetate, Merck, Netherlands). A single member of the medical staff co-ordinated all stimulation protocols, ensuring standardisation. Oocyte retrieval was performed 36 hours after the administration of choriogonadotropin alpha (Ovitrelle, Merck, Netherlands) when 2-3 follicles of 18-20mm diameter were observed by ultrasound examination, and blood 17α-oestradiol levels reached 150-200pg/ml/follicle over 18mm.

### Insemination and embryo culture techniques

All oocytes in the present project were treated with standard insemination techniques 3 hours after oocyte retrieval (60 minutes after removal of the cumulus complex). A single team of biologists co- ordinated all biological work, ensuring that both culture protocols and embryo assessment were standardised. Oocytes were processed for ICSI using commercial IVF medium (Vitrolife, Gothenberg, Sweden) pre-equilibrated to 37°C and 6% CO_2_ . Zygote quality was scored 16-17 hours after ICSI. Embryo quality on days 2 and 3 was assessed at 24 hour intervals after zygote assessment following Alpha scientist criteria (Alpha scientists, 2011), and embryo quality on days 5 and 6 were assessed using Gardner Criteria (Gardner et al., 2000).

### Cryopreservation and thaw protocols

Embryos were cryopreserved at the blastocyst stage, with a minimum quality 3CC as assessed using Gardner criteria (Gardner et al., 2000). Embryos were cryopreserved by the Kitazato vitrification technique (Kitazato, Tokyo, Japan) using Cryotop devices (Kitazato, Tokyo, Japan). Embryos were thawed using the Kitazato protocol (Kitazato, Tokyo, Japan) and cultured in IVF media (Vitrolife, Gothenberg, Sweden) for at least 60 minutes before further treatment.

### Embryo biopsy and preimplantation genetic screening techniques

Embryos were biopsied to remove a sample of the trophectoderm (McArthur et al., 2005) either prior to cryopreservation (standard preimplantation genetic screening) or a minimum of 60 minutes to 24 hours after warming of vitrified blastocysts (thaw, biopsy and refreeze strategy). Embryos were placed into pH-buffered IVF culture media (Vitrolife, Gothenberg, Sweden) prewarmed to 37°C for this purpose. Trophectoderm biopsy was performed with standard biopsy microtools on a Nikon Ti-E inverted ICSI station equipped with Narishige micromanipulators (Narishige, Japan). Immediately after the biopsy procedure had been completed, the embryo was returned to culture medium preequilibrated to 37°C and 6% CO_2_ for a minimum of 60 minutes prior to cryopreservation with the Kitazato vitrification technique (Kitazato, Tokyo, Japan).

### PGT-A analysis of biopsies

Trophectoderm samples were placed into a PCR tube (Eppendorf, Hamburg, Germany) in a solution of lysis buffer (Cooper Genomics, Nottingham, UK) and sent to the genetics laboratory (Cooper Genomics, Nottingham, UK) for assessment of ploidy with Next Generation Sequencing using PGTai technology.

### Embryo transfer and the establishment of pregnancy

Embryos defined as euploid by the PGT-A technique were selected for embryo transfer in a frozen embryo transfer cycle. Estradiol valerate (Progynova, Bayer, Germany) was administered to enable development of the uterus, followed by the administration of progesterone (Cyclogest, Collins and Co, UK) to initiate the luteal phase. Embryos were thawed 5 days after the administration of progesterone and transferred on the same day. Embryo transfer followed standard protocols. The establishment of a pregnancy was considered as a positive β-hCG test of over 20 IU/l 14 days after the administration of progesterone. The implantation rate was calculated by the observation of foetal heart beats after ultrasound analysis, 8 weeks after the establishment of pregnancy.

### Statistics

All data were plotted as mean +/- standard deviation unless stated otherwise. Students’ t-test was used to calculate the significance of groups. Regression lines were calculated by the method of least squares and the significance of the regression lines was tested with the Pearson product-moment test.

The z-test with Yates correction was used to test the significance of proportions where necessary.

## Results

A total of 54 patients were treated with the TBR protocol between 2016 and 2018 at Create fertility (Group A). During the same time-period, a further 220 patients were treated with standard PGT-A protocols including biopsy of fresh blastocysts and cryopreservation post biopsy (Group B). Maternal ages were not significantly different between group A (38.1 +/- 4.1 years, n=54) and group B (40.1 +/- 3.6 years, n=220, p=0.8, Table I).

**Table I:** — Comparison of PGT-A cycles on fresh and thawed embryos.

	Group A	Group B	p
Number patients	54	220	n/a
Maternal age	38.1 +/- 4.1	40.1 +/- 3.6	0.8
Embryos analysed	110	427	
PGT-A normal embryos (%)	31 (28.2%)	76 (17.8%)	0.02
PGT-A abnormal embryos	77 (70.0%)	331 (77.5%)	n/a
PGT-A no results	2 (1.8%)	20 (4.7%)	0.17
Cycles of FET	19	26	
Embryos thawed (mean +/- sd per cycle)	21	26	
Embryos transferred (mean +/- sd per cycle)	21 (1.05 +/- 0.4)	26 (1.00 +/- 0.0)	
Clinical pregnancies	11 (52.4%)	16 (61.5%)	0.51
Ongoing pregnancies	8 (72.7%)	14 (87.5%)	0.32

In group A, a total of 205 embryos were thawed for biopsy. These included a total of 82 cleavage stage embryos and 123 blastocysts. 42 of the cleavage stage embryos reached a stage suitable for biopsy and refreeze on day 5/6 (51.2%) and 96 blastocysts were considered suitable for biopsy and refreeze between 1 and 24 hours post thaw (78.0%, Table I). In group B, a total of 627 blastocysts were biopsied from a total of 998 fertilised eggs (62.8%) between 2016 and 2018 (Table I). The blastocyst formation and recuperation rates in group A compare favourably with blastocyst development rates in group B.

In group A, a total of 110 embryos have been analysed at the present time. Of these embryos, a total of 31 embryos were noted to be euploid after biopsy (28.2%) and therefore suitable for transfer (Table I). A further 77 embryos were aneuploid (70.0%) therefore not suitable, and 2 embryos had no result obtained after the thaw, biopsy and refreeze procedure (1.8%, Table I). These data compared favourably with group B, where 427 embryos have been analysed up until the present time. Here, 76 embryos were determined to be euploid after PGT-A (17.8%), 331 embryos were aneuploid (77.5%) and 20 embryos had no result obtained (4.6%, Table I). The proportion of euploid embryos from group A was significantly greater than group B (Table I). The proportion of embryos with no results after PGT-A was not significantly different between the two groups (Table I).

A total of 19 embryo transfer procedures were performed in group A, with 21 embryos transferred in total (1.05 +/- 0.4 per ET, Table I). From these, 11 pregnancies were obtained (57.9%) (Table I). At the present time, these pregnancies include 8 clinical pregnancies (72.7%) and 3 miscarriages (27.3%). In group B, 26 embryo transfer procedures were performed, with 26 embryos transferred in total (1.00 +/- 0 per ET, Table I). A total of 16 pregnancies were obtained (61.5%) with 14 ongoing clinical pregnancies (87.5%) and 2 miscarriages (12.5%) currently (Table I).

The above data therefore demonstrates that the thaw of previously cryopreserved embryos followed by biopsy and refreezing with the vitrification technique is effective in maintaining the viability of embryos and can be used to analyse previously cryopreserved embryos with PGT-A. Embryos used in these procedures included both cleavage stage embryos (day 2 and 3) and blastocysts (day 5 and 6). Cycles of TBR with cleavage stage and blastocyst often overlap, with both cleavage stage and blastocysts being thawed, biopsied and refrozen in the same cycle of treatment. Maternal ages for cycles where cleavage stage embryos were thawed were not significantly different to that of blastocysts (41.7 +/- 3.1 years, n=18 for cleavage stage and 39.1 +/- 4.5, n=36 for blastocyst). A total of 22 day 2 and 3 cryopreserved embryos were analysed with PGT-A and 6 embryos were determined to be euploid (27.3%). A further 15 embryos had various aneuploidies and 1 embryo failed to be analysed after PGT-A. In the same period, 88 blastocyst stage embryos were analysed and 25 of these found to be normal after PGT-A (28.4%) with a further 62 abnormal embryos found and 1 with no results after PGT-A.

## Discussion

Refreezing describes the process of cryopreservation of previously frozen-thawed embryos for future use. The application of the vitrification technique has significantly improved the reliability of both, embryo freezing and the refreeze technique. Although the literature suggests that the refreezing of embryos is a promising tool for the field of assisted reproduction, practical uses for embryo refreezing have not been developed (Ludwig et al., 1999; Farhat et al., 2001; Smith et al., 2005, Kumsako et al., 2009, Lierman et al., 2014).

PGT-A testing has significantly reduced the time to pregnancy in patients attending for assisted reproduction. However, patients with cryopreserved embryos are not usually able to request PGT-A testing due to the theoretical risk that embryos do not survive the refreeze and additional thaw process; should the embryos be defined as euploid after PGT-A. This can lead to an increase in time to pregnancy in patients with cryopreserved embryos, and the extra expense of several thaw and transfer procedures.

Recently, we have shown that refreezing of embryos with the vitrification technique can successfully enable PGT-A testing of previously cryopreserved embryos; even in cases where the embryos were cryopreserved with the slow-freeze technique (Batwala et al., 2018). The present study extended this case report to test the viability of the thaw biopsy and refreeze procedure for the analysis of previously cryopreserved embryos; whether embryos were previously cryopreserved with the slow-freeze or vitrification procedure.

Our data suggests that the thaw, biopsy and refreeze protocol is perfectly feasible to use on previously cryopreserved embryos and gives results comparable to PGT-A testing on fresh embryos.

**Table II:** — Thaw, biopsy and refreeze results on cleavage stage versus blastocysts.

	Cleavage stage	Blastocysts	p
Number patients	18	36	n/a
Maternal age	41.7 +/- 3.1	39.1 +/- 4.5	0.69
Embryos tested	22	88	
PGT-A normal embryos (%)	6 (27.3%)	25 (28.4%)	0.92
PGT-A abnormal embryos	15 (68.2%)	62 (70.5%)	0.83

In the present data, the TBR procedure gave significantly greater proportions of euploid embryos than standard PGT-A testing. However, we observed that this is not an indication that the TBR protocol improves results over the standard PGT-A protocol; but is more likely to be a statistical aberration due to the differences in group sizes. Other reasons for the apparent improvement in results from TBR could be due to the fact that blastocysts that were not suitable for biopsy and refreeze due to failure to reexpand to the stage where a biopsy could be taken were eliminated from the TBR procedure; whereas these same blastocysts may have been selected for biopsy in standard PGT-A testing. Furthermore, previously cryopreserved embryos may have been frozen at any stage after fertilisation, including the zygote stage (day 1) or cleavage stages of development (days 2 or 3). Embryos cryopreserved at stages prior to the blastocyst stage are also likely to have lower rates of recuperation due to the natural loss of embryos between days 3 and 6 (Niakan et al., 2012).

In fact, the present data does not intend to compare results from TBR and standard PGT-A testing, but simply tests the validity and efficiency of the TBR procedure. Since the data shows that TBR is at least as efficient as standard PGT-A testing, we suggest that this procedure can be offered to patients wishing for PGT-A testing on previously cryopreserved embryos.
